# Persistent Endothelial Dysfunction in Post-Acute COVID-19 Syndrome: A Case-Control Study

**DOI:** 10.3390/biomedicines9080957

**Published:** 2021-08-04

**Authors:** Pasquale Ambrosino, Ilenia Calcaterra, Antonio Molino, Pasquale Moretta, Roberta Lupoli, Giorgio Alfredo Spedicato, Antimo Papa, Andrea Motta, Mauro Maniscalco, Matteo Nicola Dario Di Minno

**Affiliations:** 1 Istituti Clinici Scientifici Maugeri IRCCS, 27100 Pavia, Italy; pasquale.ambrosino@icsmaugeri.it (P.A.); pasquale.moretta@icsmaugeri.it (P.M.); antimo.papa@icsmaugeri.it (A.P.); 2 Department of Clinical Medicine and Surgery, Federico II University, 80131 Naples, Italy; ileniacalcaterra@hotmail.it; 3 Department of Respiratory Medicine, Federico II University, 80131 Naples, Italy; molinotonio@libero.it; 4 Department of Molecular Medicine and Medical Biotechnology, Federico II University, 80131 Naples, Italy; roberta.lupoli@unina.it; 5 Department of Data Analytics and Actuarial Science, Unipol Group, 40128 Bologna, Italy; spedicato_giorgio@yahoo.it; 6 Institute of Biomolecular Chemistry, National Research Council, ICB-CNR, 80078 Pozzuoli, Italy; andrea.motta@icb.cnr.it; 7 Department of Translational Medical Sciences, Federico II University, 80131 Naples, Italy

**Keywords:** COVID-19, biomarkers, endothelial function, rehabilitation, disability, exercise, outcomes

## Abstract

Background: Endothelial dysfunction has a key role in the pathogenesis of coronavirus disease 2019 (COVID-19) and its disabling complications. We designed a case-control study to assess the alterations of endothelium-dependent flow-mediated dilation (FMD) among convalescent COVID-19 patients. Methods: COVID-19 patients referred to a Pulmonary Rehabilitation Unit within 2 months from swab test negativization were consecutively evaluated for inclusion and compared to controls matched for age, gender, and cardiovascular risk factors. Results: A total of 133 convalescent COVID-19 patients (81.2% males, mean age 61.6 years) and 133 matched controls (80.5% males, mean age 60.4 years) were included. A significantly lower FMD was documented in convalescent COVID-19 patients as compared to controls (3.2% ± 2.6 vs. 6.4% ± 4.1 *p* < 0.001), confirmed when stratifying the study population according to age and major clinical variables. Among cases, females exhibited significantly higher FMD values as compared to males (6.1% ± 2.9 vs. 2.5% ± 1.9, *p* < 0.001). Thus, no significant difference was observed between cases and controls in the subgroup analysis on females (6.1% ± 2.9 vs. 5.3% ± 3.4, *p* = 0.362). Among convalescent COVID-19 patients, FMD showed a direct correlation with arterial oxygen tension (rho = 0.247, *p* = 0.004), forced expiratory volume in 1 s (rho = 0.436, *p* < 0.001), forced vital capacity (rho = 0.406, *p* < 0.001), and diffusing capacity for carbon monoxide (rho = 0.280, *p* = 0.008). Overall, after adjusting for major confounders, a recent COVID-19 was a major and independent predictor of FMD values (β = −0.427, *p* < 0.001). Conclusions: Post-acute COVID-19 syndrome is associated with a persistent and sex-biased endothelial dysfunction, directly correlated with the severity of pulmonary impairment.

## 1. Introduction

The novel severe acute respiratory syndrome coronavirus 2 (SARS-CoV-2) first emerged in December 2019 [1], culminating in a worldwide health emergency with a pandemic declaration in March 2020 [2]. SARS-CoV-2 may be responsible for the coronavirus disease 2019 (COVID-19) [3], a syndrome with a plethora of clinical manifestations, ranging from flu-like symptoms to severe complications necessitating intensive care unit (ICU) admittance [4].

Although negativized, convalescent COVID-19 patients may experience fatigue, muscular weakness, and a decline in quality of life [5], with a persistent pulmonary impairment potentially lasting for months after the acute phase [6]. Moreover, severe cardiovascular (CV) and thromboembolic complications have also been reported among COVID-19 survivors [7,8]. Overall, given the high proportion of patients experiencing persistent clinical manifestations, the new paradigm of a “post-acute COVID-19 syndrome” has been introduced [6], thus highlighting the need of interdisciplinary post-acute care and personalized rehabilitation programs [9,10,11]. However, the pathophysiological mechanisms underlying such persistent manifestations and disabling complications have not been fully elucidated [12].

Growing evidence suggests that endothelial dysfunction may play a pivotal role in the pathogenesis of COVID-19 and its clinical manifestations [13]. SARS-CoV-2 can directly infect vascular endothelial cells (ECs) [14], with subsequent systemic endotheliitis and cellular apoptosis [15]. Furthermore, inflammatory cytokines are able to bind specific receptors on ECs surface [16], thus increasing platelet activation and leukocyte adhesion while reducing nitric oxide (NO) bioavailability [16,17]. It has been suggested that a residual activation of the immune system following the acute phase may be related to a persistent endothelial dysfunction during convalescence [18]. Accordingly, the European Society of Cardiology stressed the need for the clinical assessment of endothelial function in post-acute COVID-19 patients for monitoring and early detection of long-term CV outcomes [19].

Among the methods proposed for testing endothelial function in humans [20], flow-mediated dilation (FMD) is a non-invasive and cost-effective approach [21], being recognized as a valid substitute indicator of subclinical atherosclerosis [21] and coronary artery endothelial function [22]. Moreover, FMD is considered able to independently predict CV events [23], thus affording additional prognostic information along with conventional CV risk factors.

Therefore, we designed a case-control study to evaluate endothelium-dependent FMD among convalescent COVID-19 patients compared to non-COVID-19 controls matched for age, gender, and major CV risk factors. Moreover, we planned to perform a literature review of the physiopathological mechanisms potentially underlying our results.

## 2. Materials and Methods

### 2.1. Study Population

From December 2020 to June 2021, convalescent COVID-19 patients admitted to ICS Maugeri IRCCS, Telese Terme, Benevento, Italy for pulmonary rehabilitation were consecutively screened for study entry. Inclusion criteria were the following: age ≥ 18 years; recent SARS-CoV-2 positivity detected in nasopharyngeal swab specimens by reverse transcription polymerase chain reaction (RT-PCR); severe-to-critical COVID-19 according to the World Health Organization (WHO) criteria [24]; swab test negativization within the past 2 months (at least two negative tests, spaced 1 week apart); written informed consent signature. Exclusion criteria were an active solid tumor or hematological malignancy; a history of lung surgery; any major surgery within the past 6 months; cardio- or cerebrovascular events during the 6 months prior to the examination; any condition associated with poor compliance with the study protocol or inadequate understanding of the study procedures. Controls without a history of COVID-19, matched for age, gender, and major CV risk factors were selected from an historical cohort of subjects undergoing FMD assessment from January 2018 to July 2019. Patients with missing data for the outcome of interest were excluded from the study.

Wherever appropriate and applicable, this study was reported following the Strengthening the Reporting of Observational Studies in Epidemiology (STROBE) recommendations to limit known sources of bias [25]. The protocol was approved by the Ethics Committee of IRCCS Fondazione Pascale, Naples, Italy on 16 December 2020 (reference number 11/20-Maugeri-del-Registro), in accordance with ethical guidelines of the 1975 Declaration of Helsinki.

### 2.2. Study Protocol and Procedures

After informed consent signature, the main demographic and clinical information pertaining to the acute phase of COVID-19, pulmonary function, exercise performance, comorbidities, and ongoing treatments were collected for all convalescent COVID-19 patients. For control subjects, data were extracted from an electronic patient database (irreversibly anonymized) according to the same exclusion criteria as those of the convalescent COVID-19 cohort.

Following the National Cholesterol Education Program (NCEP) recommendations [26], hypercholesterolemia and hypertriglyceridemia were defined by total cholesterol ≥ 200 mg/dL and triglycerides ≥ 150 mg/dL, respectively. Visceral obesity was diagnosed when abdominal circumference was ≥102 cm in men and ≥88 cm in women. Study subjects were considered hypertensive if systolic blood pressure (SBP) was ≥ 30 mmHg and/or diastolic blood pressure (DBP) was ≥85 mmHg. Impaired fasting glucose (IFG) was defined by a fasting glucose ≥ 100 mg/dL.

According to the European Respiratory Society guidelines [26], arterial blood samples were tested for oxygen (PaO_2_) and carbon dioxide tension (PaCO_2_) in all convalescent COVID-19 patients at admission, using a blood gas analyzer (ABL 825^®^ FLEX BGA, Radiometer Medical Aps, Copenhagen, Denmark). Forced expiratory volume in 1 s (FEV_1_), forced vital capacity (FVC), and diffusing capacity for carbon monoxide (DLCO) were also measured with an automated instrument (Vmax^®^ Encore, Vyasis Healthcare, Milan, Italy), following the protocols of the American Thoracic Society and the European Respiratory Society [27,28,29]. FEV_1_, FVC, and DLCO were expressed both as numerical values and percentages of predicted values (FEV_1_%, FVC% and DLCO%, respectively).

The COPD Assessment Test (CAT) [30] and the Barthel scale [31] were also administered to all convalescent COVID-19 patients to determine the impact of the disease on the level of functioning and activities of daily living. Exercise capacity was tested by measuring the six-minute walking distance (6MWD) [32].

### 2.3. Brachial Artery Flow-Mediated Dilation (FMD)

Procedures for evaluating vascular reactivity parameters have been detailed elsewhere [30]. In brief, patients undergoing FMD examination were requested to abstain from food, tobacco, alcohol, and caffeine for no less than 12 h prior to the test. FMD was evaluated after ≥10 min of rest. The patient was in a supine horizontal position, with the right arm at 90 degrees abduction in the frontal plane and a blood pressure cuff positioned on the forearm. The test was carried out by a trained operator, who was blinded to the clinical and functional status of the patients. Following the recommendations of the International Brachial Artery Reactivity Task Force [31], brachial artery diameter (BAD) and blood flow velocity were monitored for 10 min with an ultrasound equipment and a 10 MHz linear probe positioned above the elbow crease. After 1 min of basal assessment, the cuff on the lower arm was inflated for 5 min to 70 mmHg above the SBP. Following cuff deflation, BAD and flow velocity were monitored for the next 4 min. An automatic edge detection software (Cardiovascular Suite^®^, FMD studio, QUIPU Srl, Pisa, Italy) registered in Europe as a medical device (MED 31116) was used for a real-time calculation of parameters of vascular reactivity.

The main outcome measure was FMD, representing the percent (%) change in BAD from baseline to the maximum value registered during reactive hyperaemia induced by forearm ischemia [31]. Post-ischemic reactive hyperemia was measured as the total area under the curve (AUC) of the shear rate after deflation [32,33], with shear rate being calculated as 4 × blood velocity/BAD [34]. Given the rest period before starting the test and the initial preparation procedures, the overall assessment took ≈ 20 min per patient.

### 2.4. Statistical Analysis

Statistical analyses and case-control matching were performed with the IBM SPSS 22.0 system (SPSS Inc., Chicago, IL, USA). Continuous data and categorical variables were expressed as mean ± standard deviation (SD) and relative frequencies, respectively. The paired samples *t*-test was used to compare continuous variables. To compare means in case of a skewed non-Gaussian distribution, we resorted to the Mann–Whitney U test. The relationship between continuous variables was evaluated using the Spearman’s correlation coefficient (rho). A linear regression analysis (stepwise method) was used to adjust for any potential confounder and to identify predictors. Statistical significance was defined by a *p* < 0.05.

Subgroup analyses were planned according to the main demographic and clinical characteristics (i.e., age, gender, hypertension, dyslipidemia, diabetes, smoking habit, obesity, history of CV events) and to the number of major vascular risk factor. Given the recognized pleiotropic effect and the potential impact on endothelial function of lipid-lowering therapies [33,35], we performed a subgroup analysis considering the presence of an ongoing statin treatment.

### 2.5. Sample Size

Based on the results of a previous report [36], a sample of 108 convalescent COVID-19 patients was needed to detect an expected absolute difference ≥ 2% in FMD between cases and controls, with a power of 99% and an α error ≤ 1%. To account for possible dropouts or protocol adherence issues, we planned to screen at least 150 convalescent COVID-19 patients and as many as controls for inclusion.

## 3. Results

### 3.1. Subjects

As reported in Figure S1, of 154 convalescent COVID-19 patients screened for eligibility, 16 (10.4%) were ineligible for protocol adherence issues and 2 (1.3%) refused to participate in the study. A total of 3 (2.2%) out of the 136 eligible patients were not considered because FMD measurement failed due to poor patient compliance in the study procedure.

A total of 133 convalescent COVID-19 patients (81.2% males, mean age 61.6 years) and 133 matched non-COVID-19 controls (80.5% males, mean age 60.4 years) were included in the final analysis. Table 1 reports the baseline clinical and demographic characteristics of patients and controls. No significant difference in age, gender, presence of CV risk factors, and ongoing CV therapies was documented between cases and matched controls.

The study sample of convalescent COVID-19 patients consisted of middle-aged subjects with a recent history of severe (30.8%) or critical COVID-19 (69.2%). The mean time from swab negativization was 16.7 ± 18.5 days. Most patients (70.7%) were transferred from an acute care setting after hospitalization with a mean length of stay of 25.4 ± 11.5 days. A total of 27.1% of patients had been mechanically ventilated during the acute phase, while 26.5% had received high-flow oxygen therapy. Patients were obese in 27.1% of cases. Hypertension was reported by 51.1% of convalescent COVID-19 patients. The prevalence of diabetes was 15.8% and current smokers were 9.0%. A history of cardiac events or stroke was found in 14.3% of convalescent COVID-19 patients.

### 3.2. Changes in FMD and Measures of Vascular Reactivity

A significantly lower FMD was documented in convalescent COVID-19 patients as compared to controls (3.2% ± 2.6 vs. 6.4% ± 4.1 *p* < 0.001), without any difference in BAD (3.9 mm ± 0.6 vs. 4.1 mm ± 0.8. *p* = 0.145) or AUC (58,915.2 ± 35,982.2 vs. 56,799.9 ± 55,596.8, *p* = 0.713).

**Figure 1:**
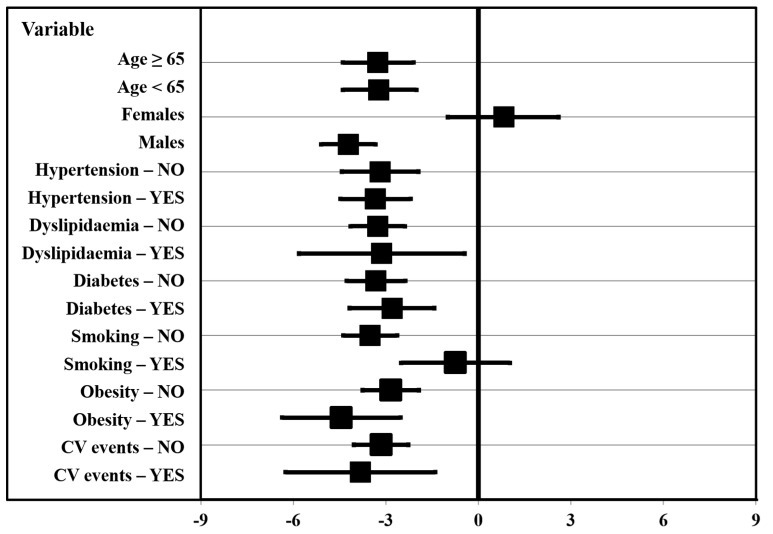
Difference in flow-mediated dilation (FMD) between convalescent coronavirus disease 2019 (COVID-19) patients and controls stratified according to demographic and clinical characteristics. CV: Cardiovascular.

No difference in FMD values was observed between severe and critical patients (3.2% ± 2.7 vs. 3.1% ± 2.4, *p* = 0.878), and no correlation was observed between FMD and the length of in-hospital stay (rho = 0.033, *p* = 0.704). When stratifying the study population according to the main demographic and clinical characteristics, differences in FMD were consistently confirmed for most variables. Moreover, we found that the difference in FMD between convalescent COVID-19 patients and controls was confirmed both in statin users (3.2% ± 2.4 vs. 8.3% ± 4.8, *p* < 0.001) and non-users (3.1% ± 2.6 vs. 6.9% ± 3.8, *p* < 0.001). In contrast, this difference was no longer significant when specifically stratifying the study population according to gender and smoking habit (Figure 1).

In particular, among convalescent COVID-19 patients, female subjects exhibited significantly higher FMD values as compared to males (6.1% ± 2.9 vs. 2.5% ± 1.9, *p* < 0.001). Thus, no significant difference was observed in FMD values between convalescent COVID-19 patients and controls when specifically analyzing the female population alone (6.1% ± 2.9 vs. 5.3% ± 3.4, *p* = 0.362). In this gender subgroup analysis, we found no difference in the distribution of the main demographic variables and vascular risk factors (Table S1).

In convalescent COVID-19 patients, similar FMD values were observed when comparing non-smokers and smokers (3.2% ± 2.6 vs. 2.4% ± 1.6, *p* = 0.156), whereas smoking habit negatively influenced FMD values in the control group (6.7% ± 4.1 vs. 3.2% ± 2.5, *p* < 0.001). Thus, no significant difference was observed in FMD values between cases and controls with a smoking habit (2.4% ± 1.6 vs. 3.2% ± 2.5, *p* = 0.392).

**Figure 2:**
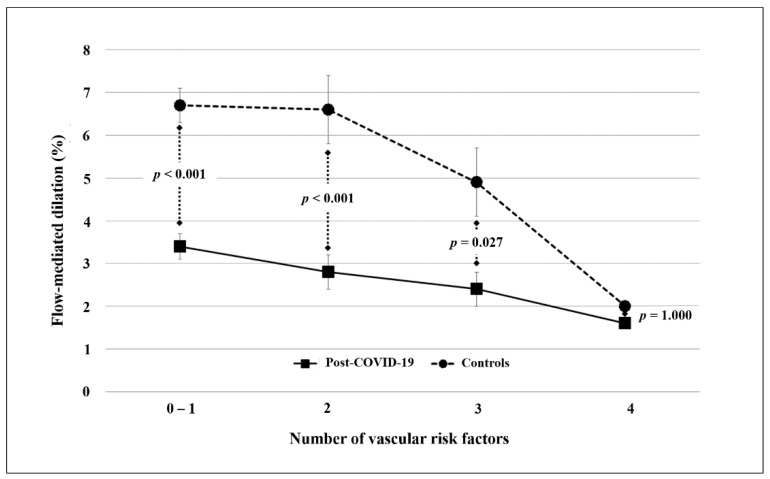
Difference in flow-mediated dilation (FMD) between convalescent coronavirus disease 2019 (COVID-19) patients and controls stratified according to the number of vascular risk factors.

The difference in FMD values was substantially confirmed when stratifying the study population according to the number of vascular risk factors (from ≤1 to 3). In contrast, in the presence of > 3 concomitant risk factors, the difference in FMD values between cases and controls was no longer significant (Figure 2).

**Figure 3:**
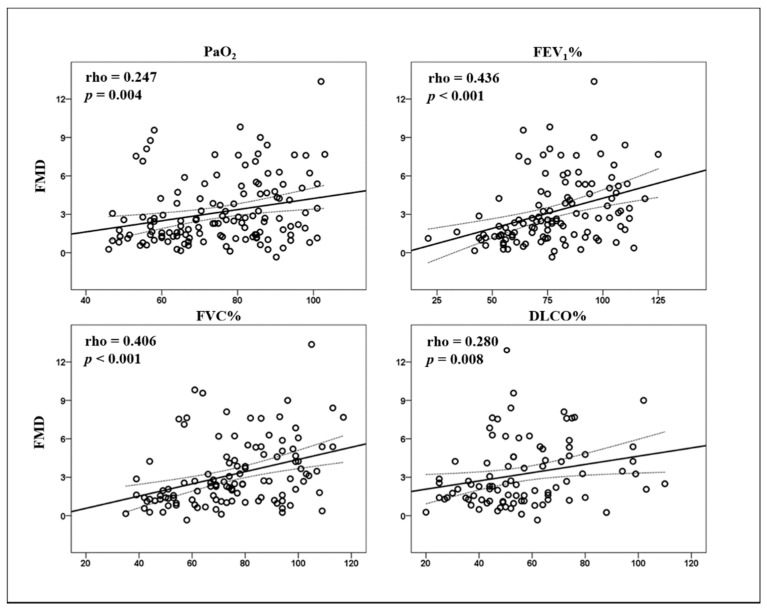
Scatter plot of Spearman’s correlations between flow-mediated dilation (FMD) and arterial oxygen tension (PaO_2_), forced expiratory volume in 1 s (FEV_1_%), forced vital capacity (FVC%), and diffusing capacity for carbon monoxide (DLCO%) in convalescent coronavirus disease 2019 (COVID-19) patients.

Among convalescent COVID-19 patients, FMD values showed a significant correlation with PaO_2_ (rho = 0.247, *p* = 0.004), FEV_1_% (rho = 0.436, *p* < 0.001), FVC% (rho = 0.406, *p* < 0.001), and DLCO% (rho = 0.280, *p* = 0.008) (Figure 3).

After adjusting for gender, age, hypertension, hypercholesterolemia, diabetes, smoking habit, obesity, and previous CV events, the correlations between FMD and these respiratory parameters were confirmed (Table S2). Overall, after adjusting for the same variables, a recent COVID-19 was a major and independent predictor of FMD values (β = −0.427, *p* < 0.001).

## 4. Discussion

In this study, we documented significantly lower FMD values among convalescent COVID-19 patients within two months from negativization, confirmed when stratifying results according to the main demographic and clinical variables. In contrast, the difference in FMD between cases and controls was no longer significant when separately considering females, smokers, and participants with several CV risk factors. A direct and significant correlation between the severity of pulmonary and vascular disease was also documented among convalescent COVID-19 patients.

### 4.1. Clinical Implications

Along with the respiratory tract, SARS-CoV-2 may affect several other organs, particularly in severe disease [37]. In this regard, the epidemiologic burden of CV manifestations cannot be overlooked when planning prevention, interventional, and rehabilitation strategies for the long-term outcomes of COVID-19 [38]. A high incidence of stroke and thromboembolic complications has been reported during the acute phase [39,40], with the risk of arrhythmic and ischemic complications [41,42,43] and subsequent hemodynamic instability [44,45]. Moreover, it has been suggested that such increased CV risk may persist during convalescence [30], as confirmed by the evidence of magnetic resonance and echocardiographic alterations [41,46] with a non-negligible incidence of venous and arterial thrombotic events [47]. However, the physiopathology of these manifestations has not been fully elucidated [48].

The relationship between endothelial dysfunction and CV disease is well known [49]. Accordingly, mounting evidence suggests that a dysfunctional endothelium may be the main pathogenic mechanism of the prothrombotic state in COVID-19 [50,51,52]. Moreover, it has been hypothesized that a residual activation of the immune system following the acute phase of COVID-19 may be responsible for a persistent endothelial dysfunction during convalescence [18]. Consequently, the European Society of Cardiology stressed the need for the clinical assessment of endothelial function in post-COVID-19 patients for preventing long-term CV complications [19].

In two previous studies [53,54], significantly lower FMD values were reported in COVID-19 patients during the acute phase as compared to healthy adults, with a value of ≤3.43% predicting mortality and longer hospital stay. Similar results were also found in a pediatric population [55]. While these studies focused on the acute phase of the disease, a previous report on 27 COVID-19 patients and 9 controls suggested the presence of persistently impaired FMD three months after virus positivity was given [36]. As reported by the Authors, a major limitation of this previous report was the small sample size. Moreover, although documenting a significant difference in FMD, the values reported in this study were substantially leveled up both in cases and controls (about 8% and 10%, respectively). This may depend on the operator dependence of FMD assessment in studies not supported by an automatic edge detection software. However, despite such limitations, the high relevance of this interesting study lies in the preliminary information of a persistent endothelial dysfunction following the acute phase.

To the best of our knowledge, ours is the first and largest study on convalescent negativized moderate-to-severe COVID-19 patients and controls matched for age, gender, and CV risk factors, assessing FMD with an automatic edge detection software. Our finding of persistently impaired FMD in the post-acute phase of COVID-19 is in line with a great amount of literature data supporting the presence of a residual CV risk after COVID-19 [41,42,43]. The clinical significance of these findings is best appreciated when we consider that each 1% absolute increase in FMD is associated with a 12% to 13% decrease in CV events [56,57]. In our study, we documented that the presence of smoking and of several traditional CV risk factors may nullify the difference between cases and controls. This may suggest that the deleterious effect of the virus on endothelial function is diluted until it disappears only in the presence of a strong factor negatively influencing endothelial function, namely cigarette smoking [58], and only when a large number of risk factors coexist. Another interesting finding of our study is the lower FMD in post-COVID-19 male patients when compared to females, with female gender leveling up FMD values and nullifying the difference between cases and controls. The literature evidence of a better prognosis and a lower incidence of CV outcomes among female COVID-19 patients is consistent with the latter result [59,60]. Finally, in line with the previous evidence of a direct and persistent correlation between the severity of pulmonary and vascular disease in this clinical setting [30], we also found that FMD values directly correlated with pulmonary function tests in our post-COVID-19 population. Of particular interest is the correlation between FMD and DLCO%, which may be justified by the functional and structural role of the endothelium within the alveolar–capillary barrier [61].

### 4.2. Physiopathology of Endothelial Dysfunction and Gender Differences in COVID-19

Overall, our findings are consistent with a growing body of evidence suggesting that ECs are a preferential target of SARS-CoV-2 [62]. It has been shown that SARS-CoV-2 is able to infect ECs using the angiotensin converting enzyme 2 (ACE2) receptor, with subsequent endotheliitis and ECs apoptosis [62]. The disruption of vascular integrity due to direct viral infection and immune-mediated inflammation leads to the exposure of the thrombogenic basal lamina and the activation of the clotting cascade [50]. Interestingly, it has been demonstrated that the ACE2 gene is located in the Xp22.2 region of the X chromosome and is recognized as an escape gene [63]. These genes appear to be protected from the repressive chromatin modifications associated with X inactivation [64]. This accounts for a gender-related difference of expression, with females potentially having a “double dose” of ACE2 [65]. Moreover, estrogens have shown regulatory activity on different components of the rennin-angiotensin system, being able to up-regulate the expression of ACE2 [66]. Overall, both genetic and hormonal factors could lead to the ACE2 over-expression among females [66,67]. This potentially compensates for the virus-induced down-regulation of ACE2, due to the endocytosis of the enzyme along with the viral particles [68] and to the up-regulation of a disintegrin and metalloproteinase 17 (ADAM17) deputized to the proteolytic cleavage of ACE2 [69].

ACE2 cannot simply be considered as the gateway for SARS-CoV-2 to human cells [70]. A key function of this enzyme is angiotensin I and angiotensin II degradation to angiotensin_1–9_ and angiotensin_1–7_, respectively [71]. These degradation peptides have a number of counter-regulatory effects on angiotensin II [70]. It has been postulated that many clinical manifestations of COVID-19 are related to ACE2 down-regulation and subsequent angiotensin II accumulation [68]. This is confirmed by the observation of a direct correlation between COVID-19 severity and angiotensin II levels [72,73].

**Figure 4:**
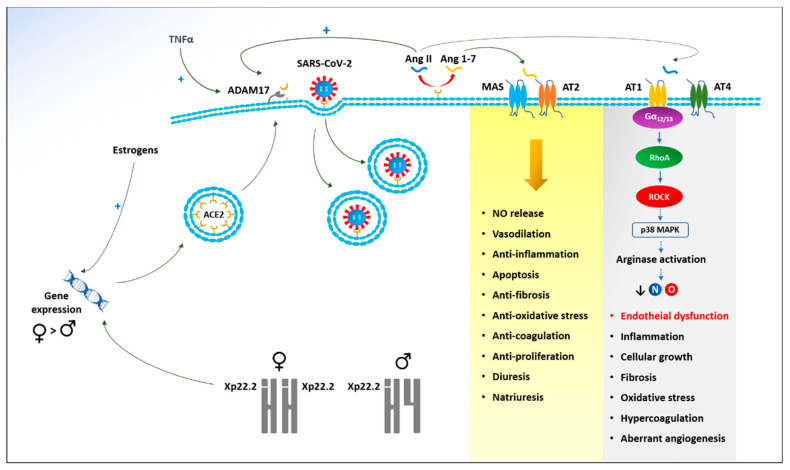
Physiopathology of endothelial dysfunction and gender differences in coronavirus disease 2019 (COVID-19). SARS-CoV-2: Severe acute respiratory syndrome coronavirus 2; TNFα: Tumor necrosis factor alfa; ADAM17: A disintegrin and metalloprotease 17; ACE2: Angiotensin converting enzyme 2; Ang II: Angiotensin II; Ang 1–7: Angiotensin_1–7_; MAS: Proto-oncogene G-protein–coupled receptor; AT1: Angiotensin receptor type 1; AT2: Angiotensin receptor type 2; AT4: Angiotensin receptor type 4; Gα_12/13_: Guanine nucleotide-binding protein alpha 12/13; RhoA: Ras homolog family member A; ROCK: Rho-associated protein kinase; p38 MAPK: p38 mitogen-activated protein kinase; NO: Nitric oxide.

Angiotensin II is an octapeptide triggering a number of functions by binding angiotensin II receptors [68]. Among them, it has been demonstrated that angiotensin II induces endothelial nitric oxide (NO) synthase dysfunction/uncoupling, which leads to decreased NO levels and increased superoxide production with oxidative stress [74]. This effect is mediated by the angiotensin II type 1 (AT1) receptor coupled to the Gα12/13 family of G proteins, with the involvement of a RhoA/Rho kinase pathway and the activation of the p38 mitogen-activated protein kinase (MAPK) [75]. Overall, this cascade mechanism results in arginase activation, reduced NO production, and endothelial dysfunction [69]. Beyond this direct effect on endothelial function, angiotensin II also stimulates inflammation by activating NF-κB, thus enhancing the transcription of inflammatory cytokines and adhesion molecules and collagen deposition [70,76]. Of high clinical importance is the over-expression and activation of endothelin-1 and plasminogen activator inhibitor-1 (PAI-1) induced by angiotensin II, accounting for its thrombogenic role [77,78] (Figure 4).

Overall, the increase in angiotensin II and the decrease in angiotensin_1–9_ and angiotensin_1–7_ result in a wide range of effects on organs and tissues, culminating in endothelial dysfunction, oxidative stress, inflammation, fibrosis, and increased CV risk. To date, females seem to have a higher ACE2-mediated CV protection for genetic and hormonal reasons. This may (at least in part) explain our findings.

### 4.3. Limitations

Some relevant limitations of the present study should be addressed. First, whereas convalescent COVID-19 patients were prospectively enrolled, control subjects were chosen from an historical cohort evaluated before the pandemic. Several issues related to the current global emergency and the organization of the Health System in our Region during the pandemic period may not allow a prospective enrollment of non-COVID-19 subjects. To partially overcome this limitation, we processed data from a matched control group according to the same exclusion criteria as those used for cases. Moreover, although FMD assessment is an operator-dependent procedure, FMD had been evaluated in controls as in cases according to the same standardized procedure [31] and using the same medical device for an automatic and real-time calculation of the vascular reactivity parameters.

Differences in BAD may represent a further confounding element, potentially affecting FMD values [79]. Interestingly, no significant difference in BAD was documented between cases and controls, thus suggesting that the reported FMD changes were likely due to differences in vascular reactivity.

Finally, we have to consider that no information on spirometry, DLCO, and blood gas analysis was available for our control subjects, thus no subgroup analysis according to pulmonary function could be performed.

## 5. Conclusions

Our results suggest the presence of persistent endothelial dysfunction among COVID-19 patients within 2 months from swab test negativization. Females may be protected from endothelial dysfunction for genetic and hormonal reasons. A periodic assessment of endothelial biomarkers, FMD, and other functional measures of endothelial activation may be useful in the follow-up of convalescent COVID-19 patients. This could help establish combined and more specific prevention, interventional, and rehabilitation strategies aimed at reducing long-term CV risk in this clinical setting.

## Figures and Tables

**Table 1:** Demographic and clinical characteristics of convalescent coronavirus disease 2019 (COVID-19) patients and controls.

Variable	Post-COVID-19	Controls	*p* Value
	133	133	
**Demographic**			
Age (Years)	61.6 ± 10.6	60.4 ± 11.5	0.380
Age > 65 Years (%)	42.9	37.6	0.453
Male Gender (%)	81.2	80.5	1.000
Smoking Habit (%)	9.0	9.0	1.000
**Acute phase COVID-19**			
WHO Class III, Severe (%)	30.8	-	-
WHO Class IV, Critical (%)	69.2	-	-
Hospitalization (%)	70.7		
Length of Hospital Stay (days)	25.4 ± 11.5	-	-
High-Flow O_2_ (%)	26.5	-	-
Mechanical Ventilation (%)	27.1	-	-
**Respiratory parameters**			
PaO_2_ (mmHg)	75.1 ± 15.0	-	-
PaCO_2_ (mmHg)	36.0 ± 4.0	-	-
FEV_1_ (L)	2.4 ± 0.7	-	-
FEV_1_ (% predicted)	77.9 ± 20.7	-	-
FVC (L)	2.9 ± 0.9	-	-
FVC (% predicted)	75.4 ± 20.0	-	-
FEV_1_/FVC	81.9 ± 9.6	-	-
DLCO (ml/min/mmHg)	11.5 ± 7.6	-	-
DLCO (% predicted)	56.4 ± 19.5	-	-
**Exercise Capacity**			
6MWD (meters)	223.3 ± 88.3	-	-
**Self-Assessment Scores**			
CAT	26.7 ± 3.4	-	-
Barthel index	72.1 ± 27.5	-	-
**Comorbidities**			
Hypertension (%)	51.1	55.6	0.539
Hypercholesterolemia (%)	9.0	10.5	0.837
Diabetes Mellitus (%)	15.8	17.3	0.869
Obesity (%)	27.1	22.6	0.478
History of Cardiovascular Events (%)	14.3	18.0	0.506
**Treatments**			
Statins (%)	29.3	17.6	0.134
Insulin (%)	12.8	10.3	1.000
Oral Hypoglycemic Agents (%)	12.0	24.1	0.137
β-Blockers (%)	31.6	41.4	0.385
Angiotensin II Receptor Blockers (%)	23.3	17.2	0.624
Antiplatelet Drugs (%)	15.3	24.1	0.275

WHO: World health organization; O_2_: Oxygen; PaO_2_: Arterial oxygen tension; PaCO_2_: Arterial carbon dioxide tension; FEV_1_: Forced expiratory volume in 1 s; FVC: Forced vital capacity; DLCO: Diffusing capacity for carbon monoxide; 6MWD: 6-min walking distance; CAT: Chronic obstructive pulmonary disease assessment test. Continuous data are presented as mean ± standard deviation. A *p* < 0.05 is statically significant.

## Data Availability

The data supporting the findings of this study are available from the corresponding authors upon reasonable request.

## Supplementary Materials

The following are available online at https://www.mdpi.com/article/10.3390/biomedicines9080957/s1, Table S1: Demographic and clinical characteristics of convalescent coronavirus disease 2019 (COVID-19) patients and controls: subgroup analysis in female gender, Table S2: Correlation between flow-mediated dilation (FMD) and pulmonary function tests (PFTs) after adjusting for gender, age, hypertension, hypercholesterolemia, diabetes, smoking habit, obesity, and previous cardiovascular events: a multivariate analysis, Figure S1: Flow chart of study participants.
