# Supplementary material for: Persistent Endothelial Dysfunction in Post-Acute COVID-19 Syndrome: A Case-Control Study
Source: Biomedicines. 2021 Aug 4;9(8):957. doi: 10.3390/biomedicines9080957 (PMC8391623; doi:10.3390/biomedicines9080957)
Supplement: Supplementary file 1 [file biomedicines-09-00957-s001.zip › biomedicines-1309967-SI.pdf]

Supplementary online material

Persistent Endothelial Dysfunction in Post-Acute COVID-19 Syndrome: A Case-Control Study.

Pasquale Ambrosino, **MD PhD**[1]* Ilenia Calcaterra, **MD**[2*]

Antonio Molino, **MD**[3] Pasquale Moretta, **PsyD**[1] Roberta Lupoli, **MD PhD**[4]

Giorgio Alfredo Spedicato, **PhD FCAS FSA CSPA CStat**[5] Antimo Papa, **MD**[1]

Andrea Motta, **PhD**[5] Mauro Manisclaco, **MD PhD**[1]** Matteo Nicola Dario Di Minno, **MD PhD**[6]**

*The two Authors equally contributed to the manuscript.
**The two Authors share co-seniorship.

[1] Istituti Clinici Scientifici Maugeri IRCCS, 27100 Pavia, Italy;
[2] Department of Clinical Medicine and Surgery, Federico II University, 80131 Naples, Italy;
[3] Department of Respiratory Medicine, Federico II University, 80131 Naples, Italy;
[4] Department of Molecular Medicine and Medical Biotechnology, Federico II University, 80131 Naples, Italy;
[5] Department of Data Analytics and Actuarial Science, Unipol Group, 40128 Bologna, Italy;
[5] Institute of Biomolecular Chemistry, National Research Council, ICB-CNR, 80078 Pozzuoli, Naples, Italy.;
[6] Department of Translational Medical Sciences, Federico II University, 80131 Naples, Italy.

Table of contents

**Table S1**	**Demographic and clinical characteristics of convalescent coronavirus disease 2019 (COVID-19) patients and controls: subgroup analysis in female gender.**
**Table S2**	**Correlation between flow-mediated dilation (FMD) and pulmonary function tests (PFTs) after adjusting for gender, age, hypertension, hypercholesterolemia, diabetes, smoking habit, obesity, and previous cardiovascular events: a multivariate analysis.**
**Figure S1**	**Flow chart of study participants.**

Table S1. Demographic and clinical characteristics of convalescent coronavirus disease 2019 (COVID-19) patients and controls: subgroup analysis in female gender.

Variable	Post-COVID-19<br>n = 133	Controls<br>n = 133	*p* Value
Age (Years)	62.8 ± 9.7	61.2 ± 11.1	0.583
Age > 65 Years (%)	48.0	42.3	0.781
Smoking Habit (%)	0	0	-
Hypertension (%)	56.0	61.5	0.779
Hypercholesterolemia (%)	4.0	0	0.490
Diabetes Mellitus (%)	8.0	15.4	0.668
Obesity (%)	24.0	19.2	0.743

Table S2. Correlation between flow-mediated dilation (FMD) and pulmonary function tests (PFTs) after adjusting for gender, age, hypertension, hypercholesterolemia, diabetes, smoking habit, obesity, and previous cardiovascular events: a multivariate analysis.

PFTs	β	p Value
PaO_2	0.191	0.008
FEV_1%	0.291	< 0.001
FVC%	0.288	< 0.001
DLCO%	0.225	0.016

PaO_2: arterial oxygen tension; FEV_1%: forced expiratory volume in 1 s (% predicted); FVC%: forced vital capacity (% predicted); DLCO%: diffusion capacity for carbon monoxide (% predicted).

Figure S1. Flow chart of study participants.

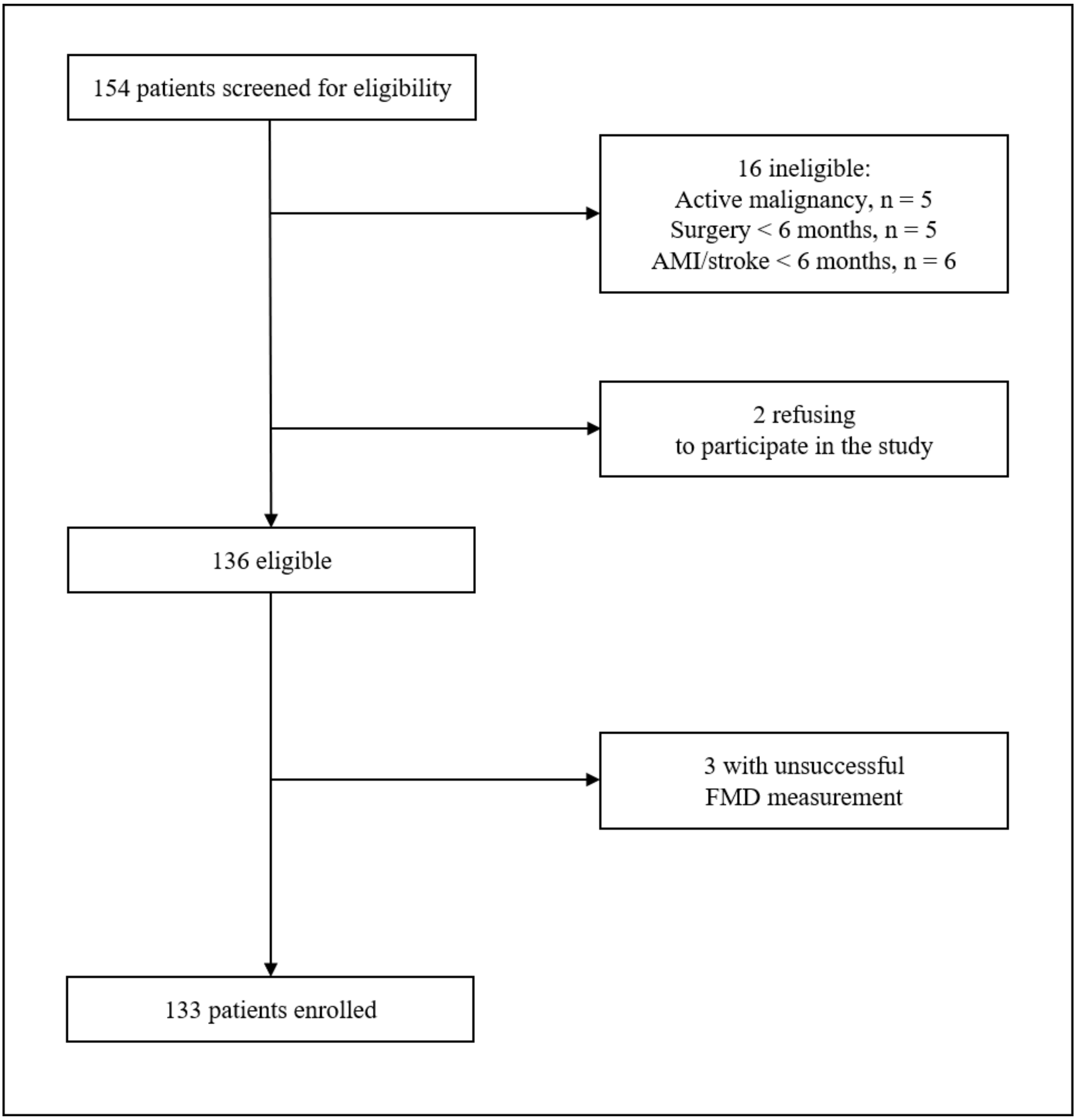

AMI: acute myocardial infarction; FMD: flow-mediated dilation.
